# Revisiting the role of cyanobacteria-derived metabolites as antimicrobial agent: A 21st century perspective

**DOI:** 10.3389/fmicb.2022.1034471

**Published:** 2022-11-18

**Authors:** Joyeeta Kar, Devde Pandurang Ramrao, Ruth Zomuansangi, C. Lalbiaktluangi, Shiv Mohan Singh, Naveen Chandra Joshi, Ajay Kumar, Kaushalendra, Suryakant Mehta, Mukesh Kumar Yadav, Prashant Kumar Singh

**Affiliations:** ^1^Department of Biotechnology, Mizoram University (A Central University), Pachhunga University College Campus, Aizawl, Mizoram, India; ^2^Centre of Advanced Studies in Botany, Institute of Science, Banaras Hindu University, Varanasi, India; ^3^Amity Institute of Microbial Technology (AIMT), Amity University, Noida, Uttar Pradesh, India; ^4^Agriculture Research Organization (ARO) - The Volcani Center, Rishon LeZion, Israel; ^5^Department of Zoology, Mizoram University (A Central University), Pachhunga University College Campus, Aizawl, Mizoram, India; ^6^Department of Botany, Mizoram University, Aizawl, India

**Keywords:** antimicrobial, bioactivity, cyanobacteria, metabolites, signaling

## Abstract

Cyanobacterial species are ancient photodiazotrophs prevalent in freshwater bodies and a natural reservoir of many metabolites (low to high molecular weight) such as non-ribosomal peptides, polyketides, ribosomal peptides, alkaloids, cyanotoxins, and isoprenoids with a well-established bioactivity potential. These metabolites enable cyanobacterial survival in extreme environments such as high salinity, heavy metals, cold, UV-B, etc. Recently, these metabolites are gaining the attention of researchers across the globe because of their tremendous applications as antimicrobial agents. Many reports claim the antimicrobial nature of these metabolites; unfortunately, the mode of action of such metabolites is not well understood and/or known limited. Henceforth, this review focuses on the properties and potential application, also critically highlighting the possible mechanism of action of these metabolites to offer further translational research. The review also aims to provide a comprehensive insight into current gaps in research on cyanobacterial biology as antimicrobials and hopes to shed light on the importance of continuing research on cyanobacteria metabolites in the search for novel antimicrobials.

## Introduction

Cyanobacteria are the photodizaotrophic, oxygen-producing microbes on earth that have gained increasing attention in natural product research. Their ubiquity in the light-exposed biosphere is based on a considerable repertoire of survival strategies for withstanding challenging environments and protecting their niches against competitors. To this end, cyanobacteria produce a wide range of secondary metabolites, often with a unique composition and specialized functions, which mediate various processes, such as chemical defence, preservation, and quorum sensing. Moreover, various metabolites with diverse bioactivities have been reported in cyanobacteria (Brilisauer et al., 2019). Some of the known cyanobacterial metabolites exhibit antiviral, antibacterial, antifungal, or herbicidal activities, promising possible applications in human health, agriculture, or industry.

The omnipresent nature of these organisms makes them excellent material for investigation by physiologists, biochemists, ecologists, molecular biologists, and pharmacists (Nagle and Paul, 1998). These metabolites’ productivity is highly species-specific and even strain-dependent (Leflaive and Ten-Hage, 2007). Because of their wide range of uses can be exploited to improve human health and sustainable living practices.

Cyanobacteria metabolites can be a gold mine for the modern healthcare industry and clinical applications. With the onset of the global pandemic we are recently facing, we are also facing the silent pandemic of antibiotic resistance. It has been estimated that 1.27 million deaths in 2019 were directly due to antibiotic-resistant infection (Murray et al., 2022). This can be attributed to the excess use of antibiotics and insufficient access to certain geographical locations (Laxminarayan, 2022). Despite the presence of vaccines, overuse of antibiotics has led to an alarming increase in antibiotic resistance among the population in recent years. Humans are susceptible to microbial pathogens such as *Escherichia coli, Staphylococcus aureus, Klebsiella pneumoniae, Pseudomonas aeruginosa, candida albicans* etc.

Furthermore, secondary infections are common in patients hospitalized with viral infections both before and after hospitalizations (Singh et al., 2011). Cyanobacteria, one of the most primitive organisms with a rich array of bioactive compounds, have evolved to protect themselves against various pathogens. Exploiting cyanobacteria to find novel antivirals and antibiotics has become more evident than ever, especially since the onset of the pandemic of Covid-19, which showed our ill-preparedness and the extreme burden on healthcare facilities across the globe. Covid-19, like other viruses, can constantly mutate, resulting in the formation of new variants such as alpha (B.1.1.7), beta (B.1.351), gamma (P.1) variants, delta (B.1.617.2) variant, Theta (P.3) variant, Lambda (C.37) variant and omicron (B.1.1.529; Vasireddy et al., 2021). These new variants have critical mutations which increase their transmissibility, infectivity, contagiousness as well as their lethality, which further necessitates the need for novel antivirals (Thakur et al., 2021).

Antibiotic-resistant microbial infections can be managed by finding novel drug discoveries, and cyanobacteria metabolites could be potential candidates (Adamson et al., 2021). For example, diterpenoid noscomin, a terpene compound isolated from *Nostoc commune*, has potent activity against pathogenic microbes like *Staphylococcus epidermidis* and *Escherichia coli* (Jaki et al., 1999). Another example is calothrixin A, an alkaloid isolated from *Calothrix* sp., which inhibits different bacteria by inhibiting bacterial RNA polymerase (Doan et al., 2000).

Considering the usability of cyanobacteria across various industries, this review aims to focus on the findings on cyanobacteria as a source of novel antimicrobials and their mechanism of action against pathogens to mitigate to issue of dwindling novel antimicrobial discovery.

## Cyanobacterial metabolites as novel antibacterial agents

**Figure 1:**
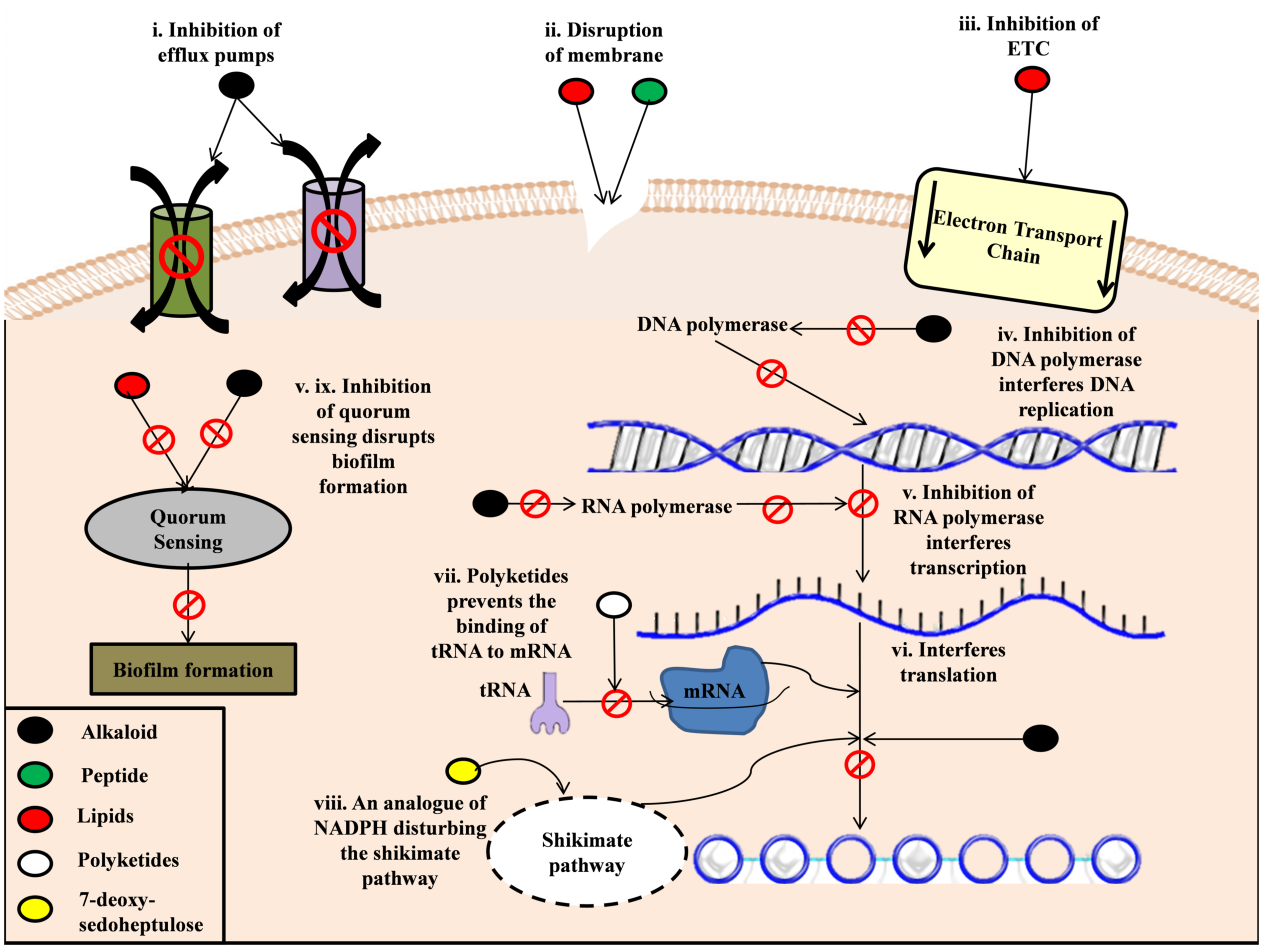
Mechanism of cyanobacterial metabolites action on bacteria. (i). Alkaloids derived from cyanobacteria inhibit the efflux pumps in bacteria (ii). Peptides and lipids of cyanobacteria disrupt the cell membrane and cause inner content leakage (iii). Lipids also inhibit the electron transport chain in bacteria disrupting the functioning of the cell (iv). Alkaloids inhibit DNA polymerase, which disrupts DNA replication (v). Alkaloids inhibit RNA polymerase, which interferes with the transcription process (vi). Alkaloids also interfere with the translation process (vii). Polyketides prevent tRNA binding to mRNA, which stops translation (iii). 7-deoxy-sedoheptulose, a sugar produced by cyanobacteria, acts as an analogue of NAPH in the Shikimate pathway, which disrupts the translation process.

The growing antibacterial resistance or bacterial antimicrobial resistance (AMR), which has been declared a silent pandemic, is one of the significant threats to public health. If left unchecked, they can prove to be far more lethal in coming years, so an urgent course of action needs to be taken to control their spread and discover newer drugs that can combat the growing resistance to the present (Murray et al., 2022). Cyanobacteria has emerged as a promising source for novel antibacterials with many antibacterial compounds. The antibacterial properties have been attributed to various combinations, namely alkaloids, terpenes, polyketides, lipids, peptides, etc. (Rojas et al., 2020). For example, Malyngolide, a polyketide isolated from *Lyngbya majuscule,* has been found to inhibit the growth of various pathogenic bacteria by inhibiting the quorum sensing system in the bacteria (Dobretsov et al., 2010; Kalia et al., 2019). Lipids like Lyngbyoic acid, pitinoic acid A, and doscadenamide A could also inhibit the quorum sensing system in *Pseudomonas aeruginosa* (Kwan et al., 2011; Montaser et al., 2011; Liang et al., 2019; Figure 1).

### Alkaloids

Alkaloids are naturally occurring nitrogen-containing compounds having a large structural diversity. Cyanobacteria-derived alkaloids, mainly indole alkaloids, have been proven to be potent antimicrobials. A large number of alkaloid compounds were extracted from *Fischerella* sp. and are found to be effective against pathogens like *Staphylococcus aureus, Mycobacterium tuberculosis, Mycobacterium smegmatis, Bacillus anthracis, Bacillus subtilis, Staphylococcus aureus, Staphylococcus epidermis*, etc. (Ghasemi et al., 2004; Mo et al., 2010). The Hapalindole group of alkaloids were also obtained from cyanobacteria like *Hapalosiphon fontinalis* and *Fischerella sp.,* which are effective against *Staphylococcus* and *Streptococcus* species (Moore et al., 1987; Mo et al., 2009a). Another example of a potent antibacterial alkaloid is Calothrixin A obtained from *Calothrix sp.,* which can inhibit *Bacillus cereus, Bacillus subtilis and Staphylococcus aureus,* respectively (Rickards et al., 1999).

### Polyketides

Polyketides are one of the most abundant secondary metabolites distributed in plants, fungi, bacteria, insects, and some marine organisms. Polyketide synthases (PKS) enzymes produce them and have wide structural diversity due to various building blocks. They are also known to have significant bioactivity and the potential for novel natural product drug discovery (Ridley and Khosla, 2009; Ma et al., 2020; Yuzawa and Kuzuyama, 2020). Polyketides like anaephenes A-C and Cylindrofridins effectively against pathogens like Methicillin Resistant *Staphylococcus aureus, Bacillus cereus, Mycobacterium tuberculosis, Streptococcus pneumonia* (Preisitsch et al., 2015b; Brumley et al., 2018). Other examples of polyketides isolated from cyanobacteria are carbamidocyclophanes A − E isolated from *Nostoc* sp. CAVN 10 (Mundt et al., 2003), carbamidocyclophanes F and G isolated from *Nostoc sp*. (Luo et al., 2014), carbamidocyclophanes H–L from *Nostoc sp.* CAVN2 (Preisitsch et al., 2015a), Nostocyclyne A from *Nostoc sp.* (Ploutno and Carmeli, 2000), and Cylindrofridins A–C from *Cylindrospermum stagnale* (Preisitsch et al., 2015b). Polyketides like anaephenes A–C and Cylindrofridins effectively against pathogens like Methicillin Resistant *Staphylococcus aureus, Bacillus cereus, Mycobacterium tuberculosis, Streptococcus pneumonia* (Preisitsch et al., 2015b; Brumley et al., 2018).

### Peptides

Peptides are abundantly extracted from cyanobacteria metabolites; many of them have been proven to be potent antimicrobials. AK-3, calophycin, hormothamnin A, lobocyclamide B, nostocyclamide, and tolybyssidin A and B are some cyclic peptides isolated from cyanobacteria. Ishida et al. isolated three potent peptide compounds; Kawaguchipeptins A and B and Norharmane-HCl [9H-pyrido (3,4-b)indole-HCl] from *Nodularia harveyana* which have potent activity against *Escherichia coli, Pseudomonas aeruginosa, Staphylococcus aureus* and *Bacillus subtilis* (Ishida et al., 1997). Laxaphycin A, Tiahuramide A-C, Hormothamnin A, [D-Leu1] MC-LR are some of the compounds extracted from cyanobacteria (Gerwick et al., 1989; Ramos et al., 2015; Dussault et al., 2016; Levert et al., 2018). A previous review by Swain et al. reported an array of peptides isolated from various cyanobacteria (Swain et al., 2017).

### Lipids

Various lipids have been isolated from cyanobacteria having antibacterial activities, such as Lyngbyoic acid, doscadenamide A, and pitinoic acid A as quorum sensing inhibitors in *Pseudomonas aeruginosa* (Carpine and Sieber, 2021) γ-linolenic acid (GLA), a potent antibacterial from Fischerella sp., was active against *Staphylococcus aureus* (Asthana et al., 2006). A lipid 2-Hydroxyethyl-11-hydroxyhexadec-9-enoate isolated from *Lyngbya sp.* is also effective against Methicillin-Resistant *Staphylococcus aureus* (MRSA). Another lipid (9Z,12Z)-9,12,15-hexadecatrienoic acid obtained from *Nostoc* sp. was effective against *Bacillus subtilis, Staphylococcus aureus* and *Micrococcus luteus* (Oku et al., 2014). Another example of cyanobacterial lipid is the Chlorosphaerolactylates family of fatty acids isolated from *Sphaerospermopsis sp.* were also found to have antibacterial activity against *Staphylococcus aureus* (Gutiérrez-del-Río et al., 2020).

### Other classes of metabolites

Terpenes and polyphenols class of compounds has also been isolated from cyanobacteria having a potent antibacterial activity (Carpine and Sieber, 2021). For example, diterpenoid noscomin isolated from *Nostoc commune* EAWAG 122b is effective against three microbes: *Bacillus cereus, Staphylococcus epidermidis*, and *Escherichia coli* (Mo et al., 2010). Polyhalogenated compounds (PHCs) like Ambigols A, B, C, D, and E are also produced by cyanobacteria which are effective against MRSA (Choi et al., 2010). Recently 7-deoxy-sedoheptulose, an unusual sugar isolated from *Synechococcus elongatus,* showed potent activity against *Anabaena variabilis,* and its mode of action was *via* mimicking 3-deoxy-D-arabino-heptulosonate 7-phosphate (DAHP), an enzyme in the shikimate pathway and inhibits the reaction mechanism pathway that leads to a decreased level of aromatic amino acids triggered by the metabolic perturbation. Further studies on other pathogenic bacteria need to be conducted using this deoxy sugar to understand their medical importance better (Brilisauer et al., 2019).

## Mechanism of action of cyanobacteria metabolites as antibacterial agents

The mechanism of action of most novel metabolites has not been explored and adequately established. However, they can be theorized using previously established studies on chemical classes and antibiotics similar to them. One of the metabolites’ most common mechanisms of action is quorum sensing inhibition. Quorum sensing is an intercellular communication system in bacteria playing an important role in virulence and biofilm formation. Berberine, an alkaloid isolated from cyanobacteria, was found to inhibit the expression of biofilm genes (Sun et al., 2019). Various lipid compounds can also inhibit the bacterial quorum sensing system of bacteria like *Escherichia coli and Pseudomonas aeroginosa* (Carpine and Sieber, 2021). Another mechanism of action is disrupting the cell membrane of the target bacteria. It was found that most lipids and peptides show antibacterial activity by disrupting membrane integrity and the subsequent cell lysis, disrupting the electron transport chain, and inhibiting important bacterial cell enzymes (Yoon et al., 2018). Different bioactive compounds also interfere with the bacterial cell’s important cellular pathways, such as the Shikimate pathway, Electron Transport chain and cell wall biosynthesis. Alkylphenols induce bacteriostasis by collapsing the proton motive force, thus inhibiting ATP synthesis and active transport (Denyer et al., 2011). A sugar isolated from *Synechococcus elongates* acts as an analogue of NADH enzyme in the Shikimate pathway, interfering with the Shikimate pathway (Brilisauer et al., 2019). In addition, the secondary metabolites of cyanobacteria are also able to the activity of various enzymes such as DNA polymerase and RNA polymerase, which affects the DNA, RNA and protein production. Alkaloids like 12-epi-hapalindole E isonitrile from *Fischerella* sp. and calothrixin A from *Calothrix sp.* showed their mode of action by inhibiting RNA polymerase independent of DNA concentration (Doan et al., 2000). Macrolides, a group of polyketides was found to interfere with aminoacyl tRNA-ribosome attachment and prevent the production of new proteins (Brumley et al., 2018).

## Cyanobacterial metabolites as antiviral agents

Viral outbreaks like Ebola, Swine influenza, and SARS-CoV-2 are a huge burden on humanity, claiming millions of human life. The emergence of new strains with mutations making them resistant to standard antiviral drugs necessitates the need for novel antivirals. Metabolites isolated from cyanobacteria have proved to be significant antivirals. Deyab and colleagues found cyanobacteria isolates: *Arthrospira platensis*, *Leptolyngbya boryana*, *Nostoc punctiforme, Oscillatoria* sp., *Leptolyngbya sp.,* and *Arthrospira platensis* isolates showed high antiviral activity against Coxsackievirus B3 and Rotavirus (Deyab et al., 2019). Cyanobacterial metabolites have also been tested as an antiviral agent against SARS-CoV2 with encouraging results (Pradhan et al., 2022). While some have been proven cytotoxic, many have shown their potential as less cytotoxic to mammalian cells, which can be used as antiviral therapeutics.

### Proteins

**Figure 2:**
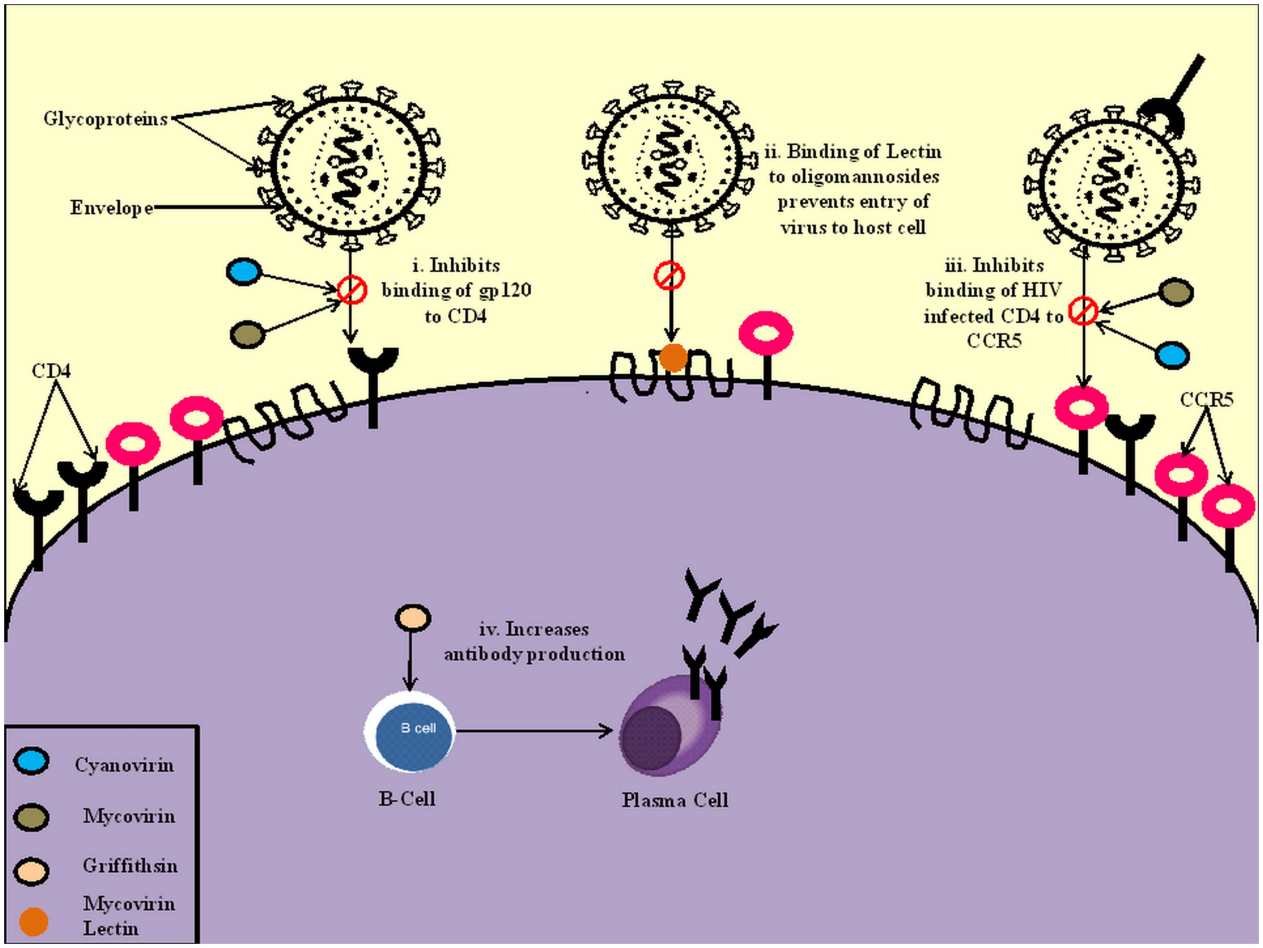
Mechanism of action of cyanobacteria on the virus. (i). Cyanovirin and Mycovirin inhibit the binding of a glycoprotein gp120 to CD4 cells, preventing the viral infection (ii). Mycovirin Lectin binds to oligomannosides on the cell wall, blocking the virus’s entry to the host cell (iii). Cyanovirin and Mycovirin block the binding of CD4 infected cells to CCR5 receptors, which prevents the progression of infection (iv). Griffithsin increases the production of antibodies and increases the reactivity of IgG.

Lectins, a carbohydrate-binding protein, is one of the most commonly isolated proteins of cyanobacteria showing promising antiviral activity (Carpine and Sieber, 2021). Cyanovirin, a lectin isolated from *Nostoc ellipsosporum,* has neutralising activity against various enveloped viruses such as HIV-1, feline immunodeficiency virus, and human herpesvirus 6 well as measles virus. A novel cyanobacterial protein, MVL, inhibited the HIV-1 Envelope-mediated cell fusion with an IC50 value of 30 nM (Bewley et al., 2004). *Oscillatoria agardhii* agglutinin (OAA), a lectin compound isolated from *Oscillatoria agardhii,* inhibited human immunodeficiency virus replication in MT-4 cells with an EC50 of 44.5 nM (Saad et al., 2022). Proteins griffithsin (GRFT) and scytovirin (SVN) isolated from cyanobacteria *Griffithsia sp.* inhibited HCV entry at nanomolar concentrations and showed significant *in vivo* efficacy in the mouse model system (Takebe et al., 2013). Cyanobacterial lectin scytovirin was demonstrated to have the ability to bind to the envelope glycoprotein of Zaire Ebola virus (ZEBOV), thus inhibiting its replication with a virus-inhibitory concentration (EC50) of 50 nM. Scytovirin is also effective against other viruses like HIV, Marburg virus and SARS-CoV2 (Garrison et al., 2014). Microvirin (MVN), isolated from *Microcystis aeruginosa,* exhibited anti-HIV activity in peripheral blood mononuclear cells with more minor cytotoxic effects than anti-human immunodeficiency virus protein cyanovirin-N, which is separated from *Nostoc ellipsosporum* (Huskens et al., 2010). *Galanthus nivalis* agglutinin (GNA) against cell culture Hepatitis C virus (HCV) was less toxic than its other lectin counterparts, *Microcystis viridis* lectin (MVL) and cyanovirin-N (CV-N), which were found to be potentially harmful due to their interaction with cellular proteins (Kachko et al. 2013a; Figure 2).

### Carbohydrates

**Figure 3:**
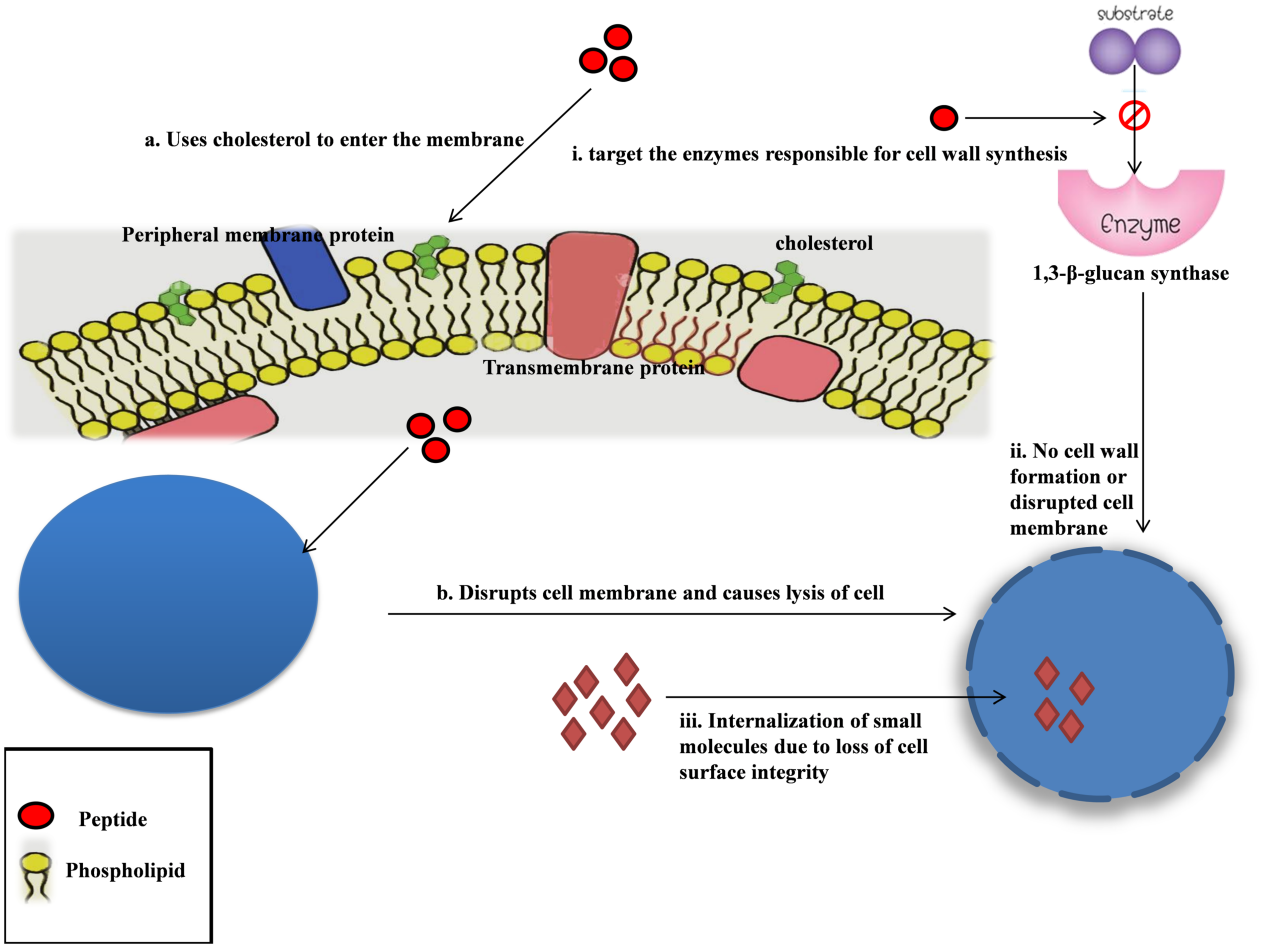
Mechanism of action of cyanobacteria as an antifungal agent. The peptides need cholesterol in the cell membrane to enter the cell, and from there on, they act by disrupting the cell membrane causing cell lysis. The peptides are hypothesized to target the enzyme responsible for cell wall synthesis. The increased internalization of molecules mainly implies a compromised cell membrane, confirming that these peptides majorly target the cell membrane.

Cyanobacteria, especially marine cyanobacteria, contain abundant polysaccharides that are effective against various viruses (Pradhan et al., 2022). Nostoflam, a polysaccharide extract of *Nostoc flagelliforme,* showed potent and broad antiviral activity against herpes simplex virus type 1 (HSV-1), human cytomegalovirus, HSV-2, and influenza A virus (Kanekiyo et al., 2005). Galactosides isolated from *Agardhiella tenera* are effective against viruses such as HSV-1, HSV-2, HIV-1, HIV-2 and Hepatitis A, respectively. A polysaccharide, Carrageenan obtained from *Chondrus, Gigartina, Hypnea, and Eucheuma* was found to be capable of blocking the entry of Dengue virus (DENV) and HPV into the host cell (Grassauer et al., 2008). Other polysaccharides such as galactans obtained from species like *Callophyllis variegata, Agardhiella tenera, Schizymenia binderi, and Cryptonemia crenulat,* also have potent antiviral activity against HSV-1, HSV-2, HIV-1, HIV-2, and DENV (Rodríguez et al., 2005). The effect of the polysaccharide Calcium Spirulan isolated from *Arthrospira platensis* is also tested against Human cytomegalovirus virus, Influenza Virus, Mumps virus, Herpes Simplex Virus-1 (HSV-1) and Human Immuno Deficiency Virus-1 with promising results (Rechter et al., 2006). Another example is Phycobiliproteins isolated from *Arthrospira platens* which have antiviral activity against Influenza A and the H1N1 virus (Chen et al., 2020; Figure 3).

### Other classes of compounds

Other compounds like alkaloids, lipids, polyphenols and polyketides have antiviral activity. Alkaloids, namely Bauerine A, B and C, effectively eradicate Herpes simplex virus-2 (Larsen et al., 1994). Other alkaloid compounds like Debromoaplysiatoxin, Anhydrodebromoaplysiatoxin, and 3-Methoxydebromoaplysia-toxin were also found to possess antiviral activity against Chikungunya virus (Gupta et al., 2014). Some lipid compounds were also found to have potent activity against Herpes Simplex Virus-1 (Chirasuwan et al., 2009). Polyketides isolated from *Trichodesmium erythraeum* were also found to be effective against the Chikungunya virus (Gupta et al., 2014).

## Mechanism of action of cyanobacteria metabolites as antivirals

Clinical studies on many cyanobacterial antivirals have been conducted with promising broad-spectrum activity, lesser cytotoxicity, and a wide range of mechanisms of action. One of the most common modes of action is preventing infection by inhibiting the binding of viral proteins to the host cell. Antiviral metabolites like Scytovirin, Cyanovirin, and Microvirin bind to viral envelope proteins of HIV like gp120, gp160 and gp41, which prevents their binding from hosting cells like CD4, thus preventing the entry of the virus (Dey et al., 2000; Bokesch et al., 2003; Huskens et al., 2010). Scytovirin also block the entry of the hepatitis C virus to the host by binding to the viral envelope glycoprotein E1 and E2 (Takebe et al., 2013). A similar mode of action was seen in Griffithsin (GRFT), a cyanobacterial lectin isolated from Griffithsia sp., where it inhibited HIV-1 by blocking the coreceptor binding process and exposing the CD4 binding site of gp120 (Alexandre et al., 2011), preventing HIV-1 capture and transmission mediated by DC-SIGN receptor (Hoorelbeke et al., 2013) and improved antibody response against virus where communization with GRFT dramatically raised the anti-gp120 IgG reactivity (Banerjee et al., 2012; Lee, 2019). Griffithsin also binds to the spike glycoproteins of MERS-CoV and SARS-CoV, preventing host cell infection (O'Keefe et al., 2010). Another mode of action is the inhibition of viral replication. This mode of action is found to be exhibited by metabolites like Calcium Spirulan, sulfoglycolipids, *Oscillatoria agardhii* agglutinin (OAA) and Scytovirin against HIV-1 and HIV-2, HSV-1 and Zaire Ebola virus (ZEBOV; Loya et al., 1998; Férir et al., 2014; Garrison et al., 2014). Scytovirin inhibits viral replication by binding to the mucin domain of the glycoprotein of the Ebola virus (Garrison et al., 2014). Lipids like Sulfoquinovosyl diacylglycerol prevents DNA replication by inhibiting HIV–reverse transcriptase and DNA polymerases (Loya et al., 1998). The metabolites also prevent further infection by binding to the host cell’s surface receptors and other host cells. An example is Mycovirin which prevents the binding of HIV-infected CD4 cells to host receptors CCR5 (Dey et al., 2000). One metabolite may have different modes of action, and the mode of action may differ in different species of viruses. For example, cyanovirin blocks the fusion pathway in HIV by blocking the binding of HIV envelope gp120 with CD4 disrupting the fusion between the host cell membrane and the virus. Not only that, but cyanovirin can also block the binding of CD4-activated gp120 to host membrane coreceptors such as CCR5. However, in the feline immunodeficiency virus, cyanovirin blocks the infection independent of CD4. They might also have other modes of action, such as interaction with another subunit of the virus envelope, such as gp41 or destabilization of the envelope (Dey et al., 2000). The potent broad-spectrum antiviral cyanobacterial-derived compounds can be explored to combat variously transmissible enveloped and non-enveloped viruses.

Arthrospira has shown potent antiviral activity in several clinical studies against HIV-1. A study showed that Arthrospira showed antiviral potential against SARS-CoV-2 using docking and *in silico* toxicity assessment. Four compounds, i.e., phycoerythrobilin, phycocyanobilin, phycourobilin, and folic acid, displayed consistent binding energies from Autodock Vina and SwissDock with low toxicity risks. Very few *in vivo* and *in vitro* studies have been conducted on cyanobacteria metabolites as potent antivirals against SARS-CoV-2. These *in silico* studies provide a new way of research advancement towards clinical studies (Petit et al., 2021). A clinical trial studied the potential use of Spirulina platensis as a nutritional supplement in adults with HIV who are undernourished in Sub-Saharan Africa. The results were promising, where the nutritional status of malnourished HIV-infected patients was improved by spirulina (Azabji-Kenfack et al., 2011). Spirulina also blocked the entry of VSV-based pseudotyped viruses (PVs) of SARS-, MERS-, and SARS-2 CoVs when preincubated with their extracts. Understanding the core mechanism of how the compounds can block the entry of viruses inside the host cells in future studies will help expand this study toward significant novel compound discovery.

This shows that broad-spectrum antiviral cyanobacterial-derived compounds can be explored to combat various transmissible enveloped and non-enveloped viruses. Furthermore, more mechanisms of action-focused studies need to be conducted on cyanobacteria metabolites as there is an evident lack of them in the current research scenario. Also, even after substantial results in pre-clinical and clinical studies, the pharmaceutical community’s antiviral properties of cyanobacterial extracts against the novel coronavirus and other human viruses seem to go unrecognized. Therefore, better approaches must be addressed to expand the research beyond academia to industry-level research.

## Cyanobacterial metabolites as antifungal agents

Owing to major challenges faced by antifungal drug development, primarily that many of the toxic molecules to the fungal organism are toxic to the hosts, too, due to the cells’ eukaryotic nature, novel antifungal discovery is slower than that of antibacterials (Seneviratne and Rosa, 2016). Nevertheless, cyanobacteria metabolites have been regarded as one of the potential sources in the discovery of antifungals with a well-established mode of action that can translate into pharmaceutical products.

*Nodularia harveyana* and *Nostoc insulare* exometabolites (norharmane and 4′4′-dihydroxy biphenyl) were effective against *Candida albicans* with a MIC value of 40 μg/ml and 32 μg/ml, respectively (Volk and Furkert, 2006). Furthermore, 10 strains of cyanobacteria were found to have bioactive compounds inhibiting *Candida albicans,* and nine inhibiting *Aspergillus flavus* in a disc diffusion assay conducted with 194 cyanobacterial strains showing a wide range of strains is capable of producing antifungal compounds, which warrants further research. In addition, the nine cyanobacteria species inhibited the growth of seven phytopathogenic fungi, which cause disease in hot pepper (*Capsicum annuum* L). Of them, two species were of Oscillatoria, two were *Anabaena*, three were Nostoc, one was Nodularia, and one *Calothrix,* which were all retrieved from rice paddy field soil (Kim, 2018).

The fatty acids isolated from *Synechocystis* sp. successfully inhibited the growth of *Candida albicans* (Najdenski et al., 2013). Similarly, the *Microcystis aeruginosa* diethyl ether extract showed potent antifungal activity against a number of *Aspergillus species, Fusarium verticillioides, Fusarium proliferatum, Penicillium verrucosum* (Marrez and Sultan, 2016). Soltani et al. (2005) reported six cyanobacterial species and one cyanobacterium species inhibited the growth of *Candida kefyr* and *Candida albicans,* respectively (Soltani et al., 2005). A polyketide tolytoxin isolated from *Scytonema* sp. and *Tolypothrix sp*. showed strong antifungal activity against a wide range of fungi such Alternaria alternate, *Aspergillus oryzae.*

*Bipolaris incurvata, Candida albicans, Saccharomyees cerevisiae, Penicillin notatum*, etc. (Patterson and Carmeli, 1992). Glycosylated lipopeptide isolated from *Tolypothrix (basionym Hassallia) sp.* exhibited antifungal activity against *Aspergillus fumigatus* and *Candida albicans* (Neuhof et al., 2005). Prasanna et al. (2008) evaluated the 35 *Anabaena* strains and reported fungicidal potential against 74 phytopathogenic fungi (Prasanna et al., 2008). Similarly, the *Lyngbya aestuarii* and *Aphanothece bullosa* showed antifungal activity against *Candida albicans* (Kumar et al., 2013). In another study conducted with Brazilian cyanobacteria crude extracts, five isolated extracts showed potent antifungal activity against *Candida albicans*. Antifungal macrolide scytophycin was found in *Anabaena* sp. HAN21/1, Nostoc sp. HAN11/1, Anabaena *cf.* cylindrica PH133, and *Scytonema* sp. HAN3/2. In the same study, Anabaena species BIR JV1 and HAN7/1, Nostoc species 6sf Calc, and CENA 219 all produced the antifungal glycolipopeptide hassallidin. Hassallidins obtained from *Nostoc calcicula, Anabaena cylindrica, and Hassallia sp.* could also inhibit different *Candida* species and *Cryptococcus neoformans* (Neuhof et al., 2005, 2006). This shows the huge potential cyanobacteria has as a potential source of antifungals having future therapeutic applications.

## Mechanism of action of antifungal activity

Most of the compounds extracted from cyanobacteria having antifungal activity are cyclic peptides in nature. Recent studies focused on their mode of action have shown that a majority of them target the cell membrane of eukaryotic cells.

Hassallidins, cyclic glycolipopeptides isolated from *Anabaena sp.,* were found to affect the function of cell membranes, resulting in lytic cell death with outer membrane disintegration and increased internalization of tiny molecules, which implies loss of cell surface integrity. It targets sterol-containing membranes, and cholesterol is necessary for them to enter the membrane (Humisto et al., 2019). Hassallidins production is not limited to *Anabaena* and *Cylindrospermopsis*; *Nostoc*, *Aphanizomenon*, and *Tolypothrix* are also known to produce them with great structural diversity and antifungal activity. They are also known to protect cyanobacteria from parasitic fungi (Vestola et al., 2014). Balticidins (A-D) are also a group of hassallidin-like lipopeptides isolated from *Anabaena cylindrica* which might have similar modes of action.

Laxaphycins A and B, first isolated from cyanobacteria *Anabaena laxa* (Patterson, 1992), have potent antifungal activity. The mode of action behind it is poorly understood, but as they are cyclic peptides in nature, it might be due to them targeting the enzymes responsible for cell wall synthesis, such as 1,3-β-glucan synthase (Zhang and Chen, 2022).

Tolytoxin, a diterpene lactone isolated from *Scytonema ocellatum* and *Scytonema mirabilei.* Has broad antifungal activity at nanomolar concentrations, and it was hypothesized that they inhibit a basic cell process exclusive to eukaryotes. Detailed modes of action on how they inhibit fungal growth are yet to be studied.

Another unrelated study showed that tolytoxin targets actin by preventing its polymerization and decreasing the number of Tunneling nanotubes (TNT), which contribute to the development of numerous diseases by facilitating the transfer of pathogens and protein aggregates (Senol et al., 2019). Furthermore, Puwainaphycins F and G, cyclic decapeptides isolated from *Cylindrospermum alatosporum,* result in necrotic cell death due to altered cell morphology and physiology, highlighting that not all antifungal peptides might be suitable for human use (Hrouzek et al., 2012). Understanding the mechanism of action behind their antifungal activity will help pinpoint which compounds can be further screened for clinical trials.

Other cyclic peptides which could be potent antifungal activity are Calophycin (Moon et al., 1992), Tolybyssidin (Jaki et al., 2001), and Schizotrin A (Pergament and Carmeli, 1994). In addition, other compounds such as alkaloid Ambiguine isonitrile (Smitka et al., 1992) and Carriebowlinol (Soares et al., 2015), terpene Scytoscalarol (Mo et al., 2009b) are also known to have potent antifungal activity. Indeed, further research on how they alter biological processes and investigations of the link between structure and activity is needed to find new application areas and potential therapeutic leads (Fewer et al., 2021).

## Challenges of using cyanobacterial metabolites

Only a few compounds of cyanobacteria have entered clinical trials, and none of the compounds has not been approved by the Food and Drug Administration (Singh et al., 2011). One of the main reasons for this is the limited knowledge of the synthesis of metabolites by cyanobacteria. The functions and regulations of the enzymes involved in the cellular pathways and biosynthetic processes were only partially known, thus complicating the use of genetic engineering techniques to produce more metabolites (Jones et al., 2009). Further investigation is also required to understand the exact mechanism of the bioactive compounds to make them feasible for pharmaceutical use (Xu et al., 2016). Another problem is the stability and bioavailability of the bioactive compounds. For example, the cyanobacterial peptides are highly unstable and require different stabilising strategies like replacing amino acids with other amino acids more resistant to hydrolysis, structural restriction, cyclization or stapling (Skowron et al., 2019). Newer strategies will greatly improve the stability and bioavailability of these peptides. Although some cyanobacterial metabolites showed promising antimicrobial activities, the cytotoxicity of some bioactive compounds like microcystins, saxitoxins or anatoxins raised severe concerns about deterring their use in the pharmaceutical and food industry (Gademann and Portmann, 2008).

## Conclusion

This review demonstrated the vast versatility of cyanobacteria and its metabolites in medical applications. They are rich in several bioactive compounds that can be explored to manage human health. Furthermore, with the advent of genetic engineering, it has been easier to manipulate a microorganism’s genetic makeup to our benefit. In the same way, metagenomics can be used to screen and mass-produce anexic cultures of cyanobacteria to produce relevant metabolites that we need and modify metabolic pathways so that we can increase the quantity of bioactive compounds in the cyanobacteria. Cyanobacteria, as a primitive organism, have a huge potential for the benefit of human welfare in the 21^st^ century as well as in the coming century with the scientific tools we have in hand. Even though they are a rich source of antifungals, antibacterials, and antioxidants, several roadblocks hinder their usage on a large scale. We need to find better ways to continue studying them to find strains with more usability and reproducibility so that their research can be forwarded to clinical trials and interdisciplinary research. It should not be labour and energy-intensive, and there should be enough co-products production along with the main compounds so that the capital and final product are cost-effective. With the right questions asked as well as small steps toward finding novel compounds and compiling new cyanobacterial strains from different habitats around the world and extracting their metabolites, we can take a bigger leap toward discovering compounds that will have a significant impact on the issues we are facing currently against antibiotic-resistant microbes and other pathogenic organisms.

## Author contributions

PKS, MKY, CL, and JK formulated and wrote the manuscript. While drafting, SMS, NCJ, K, SKM, and AK are critical suggestions in the manuscript. DPR, RZ, and CL designed the tables and figures. All authors contributed to the article and approved the submitted version.

## Funding

This study was supported by the Indian Council of Medical Research (ICMR), New Delhi (File No. 5/7/1770/Adhoc/NER/RBMCH-2021) to PS.

## Conflict of interest

The authors declare that the research was conducted in the absence of any commercial or financial relationships that could be construed as a potential conflict of interest.

## Publisher’s note

All claims expressed in this article are solely those of the authors and do not necessarily represent those of their affiliated organizations, or those of the publisher, the editors and the reviewers. Any product that may be evaluated in this article, or claim that may be made by its manufacturer, is not guaranteed or endorsed by the publisher.

## Supplementary material

The Supplementary material for this article can be found online at: https://www.frontiersin.org/articles/10.3389/fmicb.2022.1034471/full#supplementary-material
