# Supplementary material for: Revisiting the role of cyanobacteria-derived metabolites as antimicrobial agent: A 21st century perspective
Source: Front Microbiol. 2022 Nov 18;13:1034471. doi: 10.3389/fmicb.2022.1034471 (PMC9717611; doi:10.3389/fmicb.2022.1034471)
Supplement: Supplementary file 1 [file Table_1.DOCX]

**Table 1: Cyanobacterial-derived compounds and their antibacterial potential.** The table summarises the cyanobacteria compounds discovered and their bioactivity.

| Cyanobacteria Species | Compound Class | Antibacterial Compound | Inhibition against | MIC | References |
| --- | --- | --- | --- | --- | --- |
| *Nodularia harveyana* | Peptide | Aeruginazole DA1497  Kawaguchipeptins A and B  Norharmane-HCl (9H-pyrido (3,4-b)indole-HCl) | *Bacillus subtilis*  *Escherichia coli*  *Pseudomonas aeruginosa*  *Staphylococcus aureus*  *Escherichia coli*    *Pseudomonas aeruginosa*  *Bacillus subtilis*  *Staphylococcus aureus* | 2.2 µM  32 µg/ml  32 µg/ml  1 µg/ml  32 µg/ml  32 µg/ml  128 µg/ml  16 µg/ml | Raveh and Carmeli 2010  Ishida *et al.,* 1997  Volk and Furkert 2006 |
| *Lyngbya sp.* | Polyketide  Peptide | Malyngolide,  Tiahuramide A  Tiahuramide B  Tiahuramide C | *Bacillus subtilis*    *Staphylococcus aureus*  *Streptococcus pyogenes*  *Escherichia coli*  *Micrococcus luteus*  *Escherichia coli*  *Micrococcus luteus*  *Escherichia coli*  *Micrococcus luteus* | -  -  -  35 µM  47 µM  12 µM  29 µM  14 µM  17 µM | Cardellina *et al.,* 1979  Levert *et al.,* 2018 |
| *Hormothamnion*  *enteromorphoides* | Peptide | Hormothamnin A | *Bacillus subtilis*  *Pseudomonas aeruginosa* | > 100 µg/ml  > 100 µg/ml | Gerwick *et al.,* 1989 |
| *Hormoscilla sp.* | Polyketide | Anaephenes A  Anaephenes B  Anaephenes C | *Staphylococcus aureus*  *Bacillus cereus*  *Mycobacterium tuberculosis*  *Staphylococcus aureus*  *Bacillus cereus*  *Mycobacterium tuberculosis*  *Staphylococcus aureus*  *Bacillus cereus*  *Mycobacterium tuberculosis* | 22 µg/ml  11 µg/ml  >22 µg/ml  6.1 µg/ml  6.1 µg/ml  >24 µg/ml  22 µg/ml  22 µg/ml  >22 µg/ml | Brumley et al., 2018 |
| *Calothrix sp* | Alkaloid | Calothrixin A | *Staphylococcus aureus*  *Bacillus subtilis*  *Bacillus cereus* | 0.4 µM  16 µM  2 µM | Doan et al, 2000 |
| *Anabaena sp.* | Alkaloid Protein | Bromoanaindolone  C-Phycocyanin | *Bacillus cereus*  *Klebsiella pneumonia,*  *Staphylococcus aureus*  *Escherichia coli*  *Bacillus cureus* | 128 µg/mL  62 µg/mL  100 µg/mL  200 µg/mL  61.1 µg/mL | Volk *et al.,* 2007  (Safari et al., 2016  ; Osman et al. 2015) |
| *Oxynema thaianum* | Fatty acids | 9-Octadecenoic acid | *Escherichia coli*  *Klebsiella pneumonia* | 100 μg/ml  100 μg/ml | (Padmini et al. 2021) |
| *S. platensis*  *Oscillatoria amphigranulata* | Crude Extract |  | *Escherichia coli*  *Pseudomonas aeruginosa*  *Klebsiella pneumonia*  *Bacillus subtilis*  *Staphylococcus aureus*  *Escherichia coli*  *Pseudomonas aeruginosa*  *Klebsiella pneumonia*  *Bacillus subtilis*  *Staphylococcus aureus*  *Escherichia coli*  *Pseudomonas aeruginosa*  *Klebsiella pneumonia*  *Bacillus subtilis*  *Staphylococcus aureus* | 12.5 µg/ml  6.25 µg/ml  9.37 µg/ml  12.5 µg/ml  9.37 µg/ml  4.68 µg/ml  3.13 µg/ml  2.34 µg/ml  2.34 µg/ml  3.13 µg/ml  25 µg/ml  9.37 µg/ml  6.25 µg/ml  18.75 µg/ml  37.5 µg/ml | (Gheda and Ismail, 2020) |
| *Isochrysis galbana*  *Scenedesmus* sp. NT8c | Crude Extract | - | *Listeria monocytogenes*  *Staphylococcus aureus*  *Bacillus subtilis*  *Staphylococcus epidermis*  *Enterococcus faecalis*  *Listeria monocytogenes*  *Staphylococcus aureus*  *Bacillus subtilis*  *Staphylococcus epidermis*  *Enterococcus faecalis* | 12.67 µg/ml  20 µg/ml  19.33 µg/ml  18.33 µg/ml  19.33 µg/ml  18.3 µg/ml  17.67 µg/ml  18.33 µg/ml  16.67µg/ml  18.33 µg/ml | (Alsenani et al. 2020) |
| *Synechococcus* sp. | VOCs | Heptadecane (81.32%) and octadecyl acetate | *Salmonella sp.* | - | (do Amaral et al. 2020) |
| *Oscillatoria* sp. | Lipid | Hexadecanoic acid (66.3%), 1, 2-benzene dicarboxylic acid, and dibutyl phthalate | *Staphylococcus aureus,*  *Salmonella typhi* | - | (Nainangu et al. 2020) |
| *Sphaerospermopsis* sp. | Lipid | Chlorosphaerolactylates A  Chlorosphaerolactylates B  Chlorosphaerolactylates C  Chlorosphaerolactylates D | *Staphylococcus aureus*  *Staphylococcus aureus*  *Staphylococcus aureus*  *Staphylococcus aureus* | 2048 µg/ml  1024 µg/ml  1024 µg/ml  1024 µg/ml | (Gutiérrez-Del-Río et al. 2020) |
| *Fischerella sp.* | Alkaloid | Ambiguine K isonitrile  Ambiguine L isonitrile  Ambiguine M isonitrile  Ambiguine N isonitrile  Ambiguine O isonitrile  Ambiguine A isonitrile  Ambiguine B Isonitrile  Ambiguine C isonitrile  Ambiguine E isonitrile  Ambiguine F isonitrile  Ambiguine I isonitrile  Hapalindole G  Hapalindole H | *Mycobacterium tuberculosis*  *Bacillus anthracis*  *Staphylococcus aureus*  *Mycobacterium smegmatis*  *Mycobacterium tuberculosis*  *Bacillus anthracis*  *Staphylococcus aureus*  *Mycobacterium smegmatis*  *Mycobacterium tuberculosis*  *Bacillus anthracis*  *Staphylococcus aureus*  *Mycobacterium smegmatis*  *Mycobacterium tuberculosis*  *Bacillus anthracis*  *Staphylococcus aureus*  *Mycobacterium smegmatis*  *Bacillus anthracis*  *Mycobacterium tuberculosis*  *Bacillus anthracis*  *Staphylococcus aureus*  *Mycobacterium smegmatis*  *Bacillus anthracis*  *Staphylococcus aureus*  *Mycobacterium smegmatis*  *Mycobacterium tuberculosis*  *Bacillus anthracis*  *Staphylococcus aureus*  *Mycobacterium smegmatis*  *Mycobacterium tuberculosis*  *Bacillus anthracis*  *Staphylococcus aureus*  *Mycobacterium smegmatis*  *Mycobacterium tuberculosis*  *Mycobacterium tuberculosis*  *Bacillus anthracis*  *Staphylococcus aureus*  *Mycobacterium smegmatis*  *Mycobacterium tuberculosis*  *Bacillus anthracis*  *Staphylococcus aureus*  *Mycobacterium smegmatis*  *Mycobacterium tuberculosis*  *Bacillus anthracis*  *Staphylococcus aureus*  *Mycobacterium smegmatis* | 6.6 µM  7.4 µM  4.6 µM  23.7 µM  11.7 µM  16.2 µM  10.5 µM  29.3 µM  7.5 µM  28.5 µM  4.7 µM  25.8 µM  27.1 µM  30.9 µM  5.5 µM  48.8 µM  13.8 µM  46.7 µM  1 µM  1.8 µM  14.8 µM  3.7 µM  10.9 µM  27.8 µM  7 µM  16.1 µM  7.4 µM  59.6 µM  21 µM  3.6 µM  1.5 µM  1.4 µM  61.2 µM  13.1 µM  >128 µM  8.9 µM  59.7 µM  6.8 µM  >128 µM  >128 µM  34 µM  58.8 µM  >128 µM  39.6 µM | Mo *et al.*, 2008 |
| *Nostoc* sp. | Terpene  Polyketide | Noscomin  Comnostin A  Comnostin B  Comnostin C  Comnostin D  Comnostin E  4-hydroxy-7-methylindan-1- one | *Bacillus cureus*  *Staphylococcus epidermis* | -  - | Jaki et al., 1999 |
| *Anabaena oryzae* | Phycobiliprotein | Phycocyanin | *Staphylococcus aureus*  *Escherichia coli*  *Klebsiella pneumonia*  *Bacillus cureus* | 100 µg/ml  200 µg/ml | (Safari et al., 2016  ; Osman et al. 2015) |
| *Cylindrospermum stagnale* | Polyketides | Cylindrofridins | *Methicillin-resistant Staphylococcus aureus (MRSA)*  *Streptococcus pneumoniae* | 9 µM  17 µM | (Preisitsch, Niedermeyer, et al. 2015) |
| *M.aeruginosa* | Cyclic peptides (Cyanotoxin) | [D-Leu1] MC-LR | *M. tuberculosis* | 13.2 µM | (Ramos et al. 2015) |

**Table 2: Antiviral activity of compounds extracted from cyanobacteria.**

| Cyanobacteria Species | Compound Class | Antiviral Compound | Antiviral Activity  Against | IC_50_ | References |
| --- | --- | --- | --- | --- | --- |
| *Oscillatoria acuminate* | Protein | Novel Oscillatorial lectin | Herpes Simplex Virus-1 (HSV-1) viral replication  HSV-1 virions neutralisation | 131. 3 ng/ml  90.5 ng/ml | (Saad et al., 2022) |
| *Lyngabya confervoides* | Protein | Novel lyngabyal lectin | Herpes Simplex Virus-1 (HSV-1) | 0.084 µg/ml (After Infection)  0.167 µg/ml (Before Infection) | (El-Fakharany et al. 2020) |
| *Arthrospira platensis* | Polysaccharide | Calcium spirulan | Human cytomegalovirus virus  Herpes Simplex Virus-1 (HSV-1)  Human Immuno Deficiency Virus-1  Mumps Virus  Influenza Virus | 41 µg/ml (After Infection)  8.3 µg/ml (Before Infection)  165.2 µg/ml (After Infection)  0.9 µg/ml (Before Infection)  11.4 µg/ml (After Infection)  2.3 µg/ml (Before Infection)  92 µg/ml (After Infection)  23 µg/ml (Before Infection)  230 µg/ml (After Infection)  9.4 µg/ml (Before Infection) | (Rechter, Tanja, et al. 2006) |
| *Lyngbya majuscula, Schizothrix calcicola, Oscillatoria nigro-viridis, Trichodesmium erythaeum* | Alkaloid | Aplysiatoxins:  Debromoaplysiatoxin  Anhydrodebromoaplysiatoxin  3-Methoxydebromoaplysiatoxin | Chikungunya virus | 1.3 µM  22.3 µM  2.7 µM | (Gupta et al., 2014) |
| *Nostoc ellipsosporum cyanothece sp.* | Protein | Cyanovirin-N | Human Immuno Deficiency Virus-1 & 2 (HIV)  Simian  immunodeficiency virus (SIV)  SIV/HIV-1 virus (SHIV89.6P)  Feline  immunodeficiency virus (FIV)  Herpes Simplex virus (HSV)  Measles virus (MeV)  Ebola virus  Hepatitis virus  Influenza A virus  Bovine Viral Diarrhea Virus (BVDV)  Human Herpes Virus 6 (HHV-6)  Epstein-Barr virus (EBV)  Parainfleunza virus (Type 3) | 0.1 - 5.8 nM  7.6 nM and 2.3 nM  11 nMa  -  -  2.3 ± 0.12 µg/ml  5.3 ± 0.03 µg/ml  >1.0 mg/ml  7.1 ± 0.15 µg/ml  7.1 ± 0.03 µg/ml  5.3 ± 0.03 µg/ml  >10 µg/ml | O’ Keefe et al 2003 |
| *Scytonema varium* | Protein | Scytovirin | Zaire Ebola virus (ZEV)  Marburg virus (MARV) Angola Strain  Hepatitis C virus | 50 nM  100 nM  16.9 nM and 3.2 nM | (Garrison et al 2014; Soares et al., 2015) |
| *Microcystis aeruginosa* | Protein | Mycovirin | Human Immuno Deficiency Virus(HIV)-1  Human Immuno Deficiency Virus(HIV)-2 | 8 nM and 22 nM  >262 nM | (Ferir et al., 2014) |
| *Microcystis viridis* | Protein | *Microcystis viridis* lectin | Hepatitis C virus | 14.1-34.3 nM | (Soares *et al.,* 2015) |
| *Nostoc flagelliforme* | Polysaccharide | Nostoflan | Infleunza A virus  Herpes Simplex virus-1 (HSV-1)  Herpes Simplex virus-2 (HSV-2)  Human cytomegalovirus | 78 ± 16 µg/ml (After Infection)  96 ± 12 µg/ml (Before Infection)  0.37 ± 0.07 µg/ml (After Infection)  >100 µg/ml (Before Infection)  2.9 ± 0.36 µg/ml (After Infection)  7.7 ± 1.2 µg/ml (Before Infection)  0.47 ± 1.0 µg/ml (After Infection)  14 ± 2.5 µg/ml (Before Infection) | K. Hayashi et al., 2008; Kanekiyo et al., 2005 |

**Table 3:** Antifungal activity of chemical compounds extracted from cyanobacteria.

| Cyanobacteria Species | Compound Class | Antifungal Compound | A Inhibition against | MIC | References |
| --- | --- | --- | --- | --- | --- |
| *Fischerella sp.* | Alkaloid | Ambiguine H  Isonitrile  Ambiguine K isonitrile  Ambiguine L isonitrile  Ambiguine M isonitrile  Ambiguine N isonitrile  Ambiguine A isonitrile  Ambiguine B isonitrile  Ambiguine C isonitrile  Ambiguine E isonitrile  Hapalindole G  Hapalindole H | *Sacharomyces cerevisiae*  *Candida albicans*  *Candida albicans*  *Candida albicans*  *Candida albicans*  *Candida albicans*  *Candida albicans*  *Candida albicans*  *Candida albicans*  *Candida albicans*  *Candida albicans*  *Candida albicans* | 100 µg/ml  100 µg/ml  <0.9 μM  <1.0 μM  1.1 μM  <1.0 μM  <1.0 μM  1.7 μM  <1.0 μM  <0.9 μM  <128 μM  5.1 μM | (Al-Yousef and Amina 2021; Mo et al., 2008) |
| *Lyngbya majuscula* | Lactone  Lipopeptide  Cyclic Depsipeptide | Tanikolide  Hectochlorin  Lingbyabellin B | *Candida albicans* | 6.3 μg/ml | (Mareš et al. 2019) |
| *Nodularia harveyana* | Alkaloid | Norharmane-HCl (9H-pyrido(3,4-b)indole-HCl)  4’4’-dihydroxy biphenyl | *Candida albicans*  *Candida albicans* | 40 µg/ ml  32 µg/ ml | Volk and Furkert 2005 |
| *Nostoc insulare* | Phenol | 4,4’ –dihydroxy biphenyl | *Candida albicans* | 32 µg/ ml | Volk and Furkert 2005 |
| *Symplocastrum muelleri* | Cyclic polypeptide | Puwainaphycins F | *Candida albicans and Saccharomyces cerevisiae* | -  - | (Fewer et al., 2018) |
| *Microcystis aeruginosa* | Fatty acid | Butylated Hydroxytoluene (BHT)  Hexadecanoic acid methyl ester | *Aspergillus flavus*  *Aspergillus parasiticus*  *Aspergillus  westerdijikia*  *Aspergillus steynii*  *Aspergillus ochraceus*  *Aspergillus carbonarius*  *Fusarium verticillioides*  *Fusarium proliferatum*  *Penicillium verrucosum*  *Aspergillus flavus*  *Aspergillus parasiticus*  *Aspergillus  westerdijikia*  *Aspergillus steynii*  *Aspergillus ochraceus*  *Aspergillus carbonarius*  *Fusarium verticillioides*  *Fusarium proliferatum*  *Penicillium verrucosum* | 2.24 mg/ml  1.98 mg/ml  3.12 mg/ml  2.44 mg/ml  3.02 mg/ml  2.28 mg/ml  2.84 mg/ml  3.78 mg/ml  3.22 mg/ml  0.94 mg/ml  0.91 mg/ml  1.04 mg/ml  1.22 mg/ml  1.26 mg/ml  1.3 mg/ml  0.93 mg/ml  0.82 mg/ml  1.24 mg/ml  -  - | (Marrez and Sultan 2016) |
| *Cyanobacteria strain BCBC12-12* | Alkaloid | CarrieBowlinol  Lyngbic Acid | *Fusarium sp.,*  *Lindra thalassiae*  *Dendryphiella salina*  *Fusarium sp.,*  *Lindra thalassiae*  *Dendryphiella salina* | 0.4 µM  0.8 µM  0.5 µM  4.2 µM  5.4 µM  5.8 µM | (Soares et al. 2015) |
| *Scytonema sp, Tolypothrix sp.* | Polyketide | tolytoxin (6-hydroxy-7-O-methyl-scytophycin b) | *Alternaria alternata*  *Aspergillus oryzae*  *Bipolaris incurvata*  *Caloneetria critalarae*  *Candida albicans*  *Colletotrichum eoecodes*  *Penicillium notatum*  *Rhizoctonia solani*  *Saccharomyces cerevisiae*  *Thielaviopsis paradoxa*  *Trichophyton mentagrophytes* | 4 µM  0.5 µM  2 µM  2 µM  8 µM  4 µM  0.25 µM  0.25 µM  4 µM  1 µM  8 µM | Patterson and Carmeli 1992 |
| *Nostoc calcicula, Anabaena cylindrica, Hassallia sp.* | Glycolepopeptide | Hassallidins A  Hassallidin B | *Candida albicans*  *Candida glabrata*  *Candida guillermondii*  *Candida tropicalis*  *Candida parasilopsis*  *Candida krusei*  *Candida albicans*  *Candida glabrata*  *Candida guillermondii*  *Candida tropicalis*  *Candida parasilopsis*  *Candida krusei*  *Cryptococcus neoformans* | 4 μg/ mL  8 μg/ mL  8 μg/ mL  8 μg/mL  8 μg/mL  8 μg/mL  8 μg/ mL  16 μg/ mL  8 μg/ mL  8 μg/mL  16 μg/mL  16 μg/mL  8 μg/mL | (Neuhof et al., 2005; Neuhof et al., 2006) |
| *Anabaena cylindrica* | Lipopeptide | Balticidins A-D | *Candida albicans*  *Candida maltosa*  *Candida krusei*  *Aspergillus fumigatus*  *Microsporum gypseum*  *Microsporum canis*  *Mucor sp.* | 0.1 μg/mL | (Bui et al., 2013) |
